# Multiple Health Risk Factors in Vocational Education Students: A Systematic Review

**DOI:** 10.3390/ijerph18020637

**Published:** 2021-01-13

**Authors:** Prince Atorkey, Judith Byaruhanga, Christine Paul, John Wiggers, Billie Bonevski, Flora Tzelepis

**Affiliations:** 1 School of Medicine and Public Health, University of Newcastle, Callaghan, NSW 2308, Australia; Judith.Byaruhanga@uon.edu.au (J.B.); Chris.Paul@newcastle.edu.au (C.P.); John.Wiggers@health.nsw.gov.au (J.W.); Billie.Bonevski@newcastle.edu.au (B.B.); Flora.Tzelepis@newcastle.edu.au (F.T.); 2 Hunter New England Population Health, Hunter New England Local Health District, Wallsend, NSW 2287, Australia; 3 Hunter Medical Research Institute, Kookaburra Circuit, New Lambton Heights, NSW 2305, Australia; 4 Priority Research Centre for Health Behaviour, Faculty of Health & Medicine, University of Newcastle, Callaghan, NSW 2308, Australia

**Keywords:** multiple health risk factors, vocational education students, clustering, co-occurrence

## Abstract

Health risk factors such as tobacco smoking, inadequate fruit intake, inadequate vegetable intake, risky alcohol consumption, physical inactivity, obesity, anxiety and depression often commence during adolescence and young adulthood. Vocational education institutions enrol many students in these age groups making them an important setting for addressing multiple health risk factors. This systematic review examined (i) co-occurrence of health risk factors, (ii) clustering of health risk factors, and (iii) socio-demographic characteristics associated with co-occurrence and/or clusters of health risks among vocational education students. MEDLINE, PsycINFO, EMBASE, CINAHL and Scopus were searched to identify eligible studies published by 30 June 2020. Two reviewers independently extracted data and assessed methodological quality using the National Heart, Lung and Blood Institute Quality Assessment Tool. Five studies assessed co-occurrence and three studies clustering of health risks. Co-occurrence of health risk factors ranged from 29–98% and clustering of alcohol use and tobacco smoking was commonly reported. The findings were mixed about whether gender and age were associated with co-occurrence or clustering of health risks. There is limited evidence examining co-occurrence and clustering of health risk factors in vocational education students. Comprehensive assessment of how all these health risks co-occur or cluster in vocational education students is required.

## 1. Introduction

Noncommunicable diseases (NCDs) such as cancer, diabetes and cardiovascular diseases are the leading cause of death globally [1]. Modifiable behavioural health risk factors such as smoking tobacco, inadequate fruit consumption, inadequate vegetable intake, risky alcohol consumption, physical inactivity and obesity have consistently been found to be associated with increased risk of NCDs and mortality from such diseases [2,3,4,5]. Often, these health risk factors can co-occur or cluster with psychological distress such as depression and anxiety [6,7,8,9]. Co-occurrence of health risk factors refers to concurrent engagement in two or more health risk factors and clustering refers to the association between co-occurring health risk factors [10]. Concurrently engaging in two or more health risk factors and clustering of health risk factors increase the risk of non-communicable diseases and deaths compared to no health risk factors [2,11]. For instance, a longitudinal study that examined the combined effect of four health risk behaviours (i.e., smoking, inadequate fruit and vegetable intake, alcohol intake and physical inactivity) among British adults revealed that the risk of mortality was greater among those engaging in all four health risk behaviours compared to those engaging in one health risk behaviour [3]. Addressing multiple health risk factors may therefore maximise health benefits and result in a greater reduction in health care costs [12,13]. As part of primary preventive strategies to reduce the global chronic disease burden, the World Health Organization recommends approaches that tackle these multiple health risk factors instead of targeting single health risk factors [14].

Health risk factors often commence during adolescence and become established during early adulthood [15,16]. Vocational education institutions are therefore an important setting to address multiple health risk factors in students because most students who enrol in vocational education are adolescents or young adults [17]. Vocational education settings include trade schools, technical schools, community colleges, colleges of further education, institutes of technology, apprenticeship training, career and technical education and polytechnic institutions [18]. Vocational education settings prepare students for specific occupations in trade or craft as technicians, or in professional vocations such as dentistry [17]. This training can be offered at the secondary, post-secondary, non-tertiary, further education, or higher education level [18].

The transition into vocational education may be characterised by the individual’s growing independence that may lead to unhealthy lifestyle choices such as smoking tobacco, frequent consumption of fast foods rather than fruit and vegetables, risky consumption of alcohol, inadequate physical activity, increase in body weight, and the experience of psychological distress such as anxiety and depression [19,20,21]. Factors such as the cost of healthy foods being greater than unhealthy fast foods [22], the high cost of using the gym, busy lifestyles, cognitive-emotional factors (i.e., lack of confidence), social smoking and drinking with peers may contribute to multiple health risk factors in vocational education students [22,23,24].

Furthermore, vocational education students compared to university students are more likely to engage in health risk behaviours and to experience psychological distress [25,26,27]. This may be due to additional competing demands and/or life circumstances as vocational education students are more likely to work full time than university students, have low socio-economic status and be part of minority groups [25,28].

Two systematic reviews have examined clustering of multiple health risk behaviours [29,30], although not specifically within the vocational education setting. The systematic review by Meader and colleagues identified which risk behaviours (i.e., tobacco smoking, low fruit and vegetable intake, alcohol misuse, physical inactivity, illicit drug use, and sexual risk) cluster or co-occur and the socio-demographic factors associated with co-occurrence or clustering of health risk behaviours [30].

The systematic review by Noble and colleagues examined clustering of smoking tobacco, nutrition, alcohol, and physical activity (SNAP) health risk behaviours and socio-demographic characteristics associated with SNAP health risk clusters [29]. More than half of the studies included in this review reported clustering of smoking tobacco and alcohol use [29]. Among the five studies that examined the clustering of health risk behaviours among university students, three studies reported clustering of alcohol use and smoking tobacco [29]. None of these reviews included studies that examined obesity, depression, and anxiety. Furthermore, none of the existing reviews included studies that examined the co-occurrence and/or clustering of multiple health risk factors among vocational education students.

In relation to young adults, the existing systematic reviews on multiple health risk factors focused on university students but not vocational education students [29,30]. This is despite a substantial proportion of young adults attending vocational education settings and not universities [17], highlighting a need to also examine multiple health risk factors in this important sub-group of young adults. Therefore, a systematic review that synthesises studies investigating multiple health risk factors among vocational education students is necessary to address the existing gap in the literature and to inform the development of effective health promotion interventions targeting multiple health risk factors among vocational education students. This systematic review aims to examine among vocational education students:

- The co-occurrence of key multiple health risk factors responsible for NCDs (i.e., at least two of smoking tobacco, inadequate fruit intake, inadequate vegetable intake, risky alcohol consumption, physical inactivity, obesity, anxiety and depression);
- Clustering patterns of these health risk factors;
- Socio-demographic characteristics associated with co-occurrence of health risk factors or identified clusters.

## 2. Materials and Methods

### 2.1. Design and Registration

The systematic review was a narrative synthesis of studies registered with PROSPERO (International Prospective Register of Systematic Reviews) (registration number: CRD42019118161) and was conducted following the guidelines in the Preferred Reporting Items for Systematic Review and Meta-Analysis (PRISMA) [31].

### 2.2. Literature Search

The following electronic databases were searched: MEDLINE, PsycINFO, EMBASE, CINAHL and Scopus to identify studies published by 30 June 2020 that examined multiple health risk factors in vocational education settings. The search was undertaken using keywords and medical subject heading searches (MesH). Boolean Operators “AND” and “OR” were used to combine search terms where appropriate. “OR” was used for within group combinations while “AND” was used for between group combinations. The search was restricted to studies with human participants. The reference lists of included articles were also checked to identify other eligible articles that may not have been captured during the database search. Table 1 outlines the search strategy. 

### 2.3. Inclusion Criteria

#### 2.3.1. Type of Studies

Only quantitative studies published in English in peer-reviewed journals and published thesis or dissertations were included.

#### 2.3.2. Study Design

Studies were included if they used any of the following designs: cross-sectional, longitudinal/cohort studies or baseline data from randomised controlled trials where co-occurrence and/or clustering of any combination of smoking tobacco, fruit intake, vegetable intake, alcohol consumption, physical activity, obesity, anxiety and depression was reported.

#### 2.3.3. Participants

Studies were included if they surveyed students attending a vocational education institution only or subgroup analysis was available for the vocational education students.

#### 2.3.4. Outcomes

Studies were included if they analysed together at least two of the following outcome measures:

- Smoking tobacco: any measure assessing current tobacco smoking behaviours (e.g., cigarette smoking);
- Fruit intake: any measure assessing fruit intake (e.g., daily serves of fruits);
- Vegetable intake: any measure assessing vegetable intake (e.g., daily serves of vegetables);
- Alcohol use: any measure assessing alcohol consumption (e.g., standard drinks per day);
- Physical activity: any measure of physical activity (e.g., minutes of moderate or vigorous physical activity);
- Obesity: any measure of obesity (e.g., body mass index, waist circumference);
- Depression: any measure of depression (e.g., having low interest in doing things);
- Anxiety: any measure of anxiety (e.g., feeling nervous or on edge).

Co-occurrence of multiple health risk factors was defined as concurrent engagement in two or more health risk factors and clustering was defined as association between co-occurring health risk factors [10].

### 2.4. Study Exclusion Criteria 

Studies were excluded if they reported co-occurrence of multiple health risk factors where the percentage contained a majority (i.e., at least 4) of health risk factors not addressed by this review (e.g., illicit drug use, carrying a weapon, non-use of seat belts or crash helmets, physical fighting). Studies were also excluded if they were conducted in the vocational education setting but assessed the staff rather than the student population. Conference proceedings, non-peer reviewed articles, commentaries, protocols, systematic reviews, case control studies and non-English publications were all excluded.

### 2.5. Screening

All articles during the electronic search were exported into Endnote (Version 9, Clarivate Analytics, Philadelphia, PA, USA and duplicates removed. After deduplication, articles were exported into Covidence for title and abstract screening. Title and abstract screening was completed independently by two members (P.A. and (F.T. or J.B.)) of the review team based on the inclusion and exclusion criteria. If the eligibility of the study could not be determined during the title and abstract screening the full text of the article was obtained. Full-text screening was completed independently by two members (P.A. and (F.T. or J.B.)) of the review team. Discrepancies were resolved between the two reviewers. Reasons for exclusion during the full-text screening were recorded. Using Cohen kappa, inter-rater reliability between the two raters was k= 0.80, demonstrating substantial agreement [32].

### 2.6. Data Extraction

Data extraction was performed independently by two members of the review team (P.A. and J.B.). The reviewers discussed any discrepancies until resolved and if required consulted a third reviewer to resolve disagreements (F.T.). The following data were extracted from each of the included studies:

- Publication details: author(s), publication year, country of study and year data were collected;
- Study setting: type of vocational education setting;
- Study design: cross-sectional studies, longitudinal studies, and baseline data from randomised controlled trials;
- Sample characteristics: socio-demographic characteristics (e.g., age, gender, education, employment status, socio-economic status, marital status, country of birth, area of residence), sample size, recruitment methods used, eligibility criteria, consent rates;
- Measures: type of tobacco smoking, fruit intake, vegetable intake, alcohol use, physical activity, obesity, depression, and anxiety measures used;
- Outcomes: co-occurrence of two or more health risk factors, clustering of multiple health risk factors and socio-demographic characteristics associated with co-occurrence of multiple health risk factors and/or identified clusters.

### 2.7. Methodological Quality Assessment

The National Heart, Lung and Blood Institute (NHLBI) standardized Quality Assessment Tool for Observational Cohort and Cross-sectional studies [33] was used to assess the methodological quality of eligible studies. This quality assessment tool has 14 items with three response options (Yes, No, and other (i.e., CD, cannot determine; NA, not applicable, and NR, not reported). Quality of studies was judged as “good”, “fair” or “poor” based on the ratings of the items in the tool. This was done independently by two members (P.A. and F.T.) of the team. These two reviewers discussed any discrepancies until resolved.

### 2.8. Data Analysis and Synthesis

A systematic narrative synthesis was conducted as heterogeneity across the included studies did not allow for meta-analysis. The characteristics of studies, co-occurrence of health risk factors or clustering of health risk factors were presented using tables and narrative summaries. We followed the Guidance of the conduct of Narrative synthesis in Systematic Reviews [34]. P.A. performed all analysis and synthesis with the guidance of the other members of the review team.

## 3. Results

**Figure 1:**
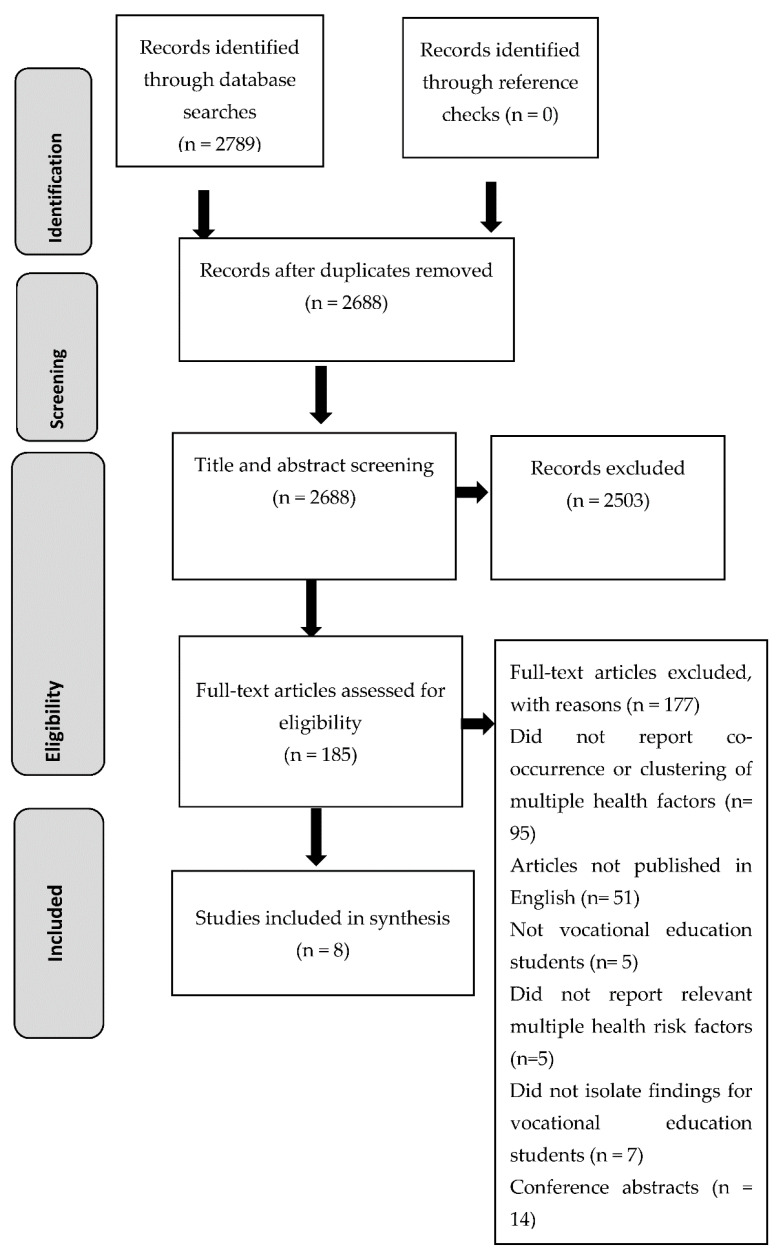
PRISMA diagram of screening and selection process.

Figure 1 presents the PRISMA diagram for screening and selection. A total of 2789 records were identified during the database search, and after duplicates were removed, 2688 records were screened. During the title and abstract screening, 2503 records were excluded leaving 185 full-text records which were assessed for eligibility (Figure 1). Overall, 177 of the full-text records were excluded and reasons for exclusion are presented in Figure 1. Eight studies [35,36,37,38,39,40,41,42] were deemed eligible and were included in the review.

### 3.1. Study Characteristics

Table 2 describes the characteristics of included studies. Two studies were conducted in the United States [37,39] and one study in each of Australia [36], the Netherlands [35], France [38], Switzerland [42], Germany [40] and United Kingdom [41]. All eight studies were published from 2007 onwards. Seven studies used a cross-sectional design [35,36,37,38,39,41,42], and one study used a longitudinal design [40]. The sample size ranged from 142 [37] to 5688 [40]. Five studies reported mean age which ranged from 17.4 years [38] to 22.75 years [37]; one study reported a median age of 18 years [42], and one study recruited participants 16 years and older [36]. Five studies [35,36,37,39,42] reported a majority of female participants whereas two studies [38,40] reported a majority of male participants. For recruitment method used, two studies recruited students using an information sheet distributed to students weeks prior to the data collection [35,36]; one study displayed posters, distributed handouts around campuses and advertised via community college newspapers and email [37]; one study recruited all adolescents who attended the National Defence and Citizenship Day (JDC) [38]; one study recruited students during a regular school lesson reserved for health education [42]; one study recruited students by sending questionnaire packs to their advisors who distributed them during their study day [41], and two studies did not report recruitment method [39,40]. Across the eight studies the response rate ranged from 66% [41] to 99.5% [39,42].

### 3.2. Combinations of Health Risk Factors Measured

Three of the studies measured two health risk factors [38,40,41]. The combinations assessed were smoking cigarettes/tobacco and drinking alcohol for all three studies. Three studies measured three health risk factors [35,37,42]. The combinations explored were alcohol drinking, depression and anxiety [37], hazardous drinking, smoking and physical inactivity and cigarette smoking, binge drinking and depression [35,42]. One study measured four health risk factors (i.e., tobacco use, alcohol use, leisure time physical activity and overweight/obesity) [39], whereas one study measured six of the health risk factors (i.e., smoking tobacco, alcohol consumption, fruit consumption, vegetable consumption, physical activity and obesity/overweight) [36]. None of the studies measured all eight health risk factors examined in this review.

### 3.3. Co-Occurrence of Multiple Health Risk Factors

Five studies reported the co-occurrence of multiple health risk factors among vocational education students [36,37,38,41,42]. Across the five studies, vocational education students’ engagement in two or more health risk factors ranged from 29% [42] to 98% [36].

### 3.4. Clustering Patterns of Health Risk Factors

Three studies examined the clustering of health risk factors [35,39,40]. Bannink et al. reported two clusters (i.e., “substance use” cluster characterised by binge drinking, cannabis use and cigarette smoking and “problem behaviour” cluster characterised by students who were delinquent, truant and incurred debts) using principal component analysis [35]. The study by Jeffries et al. reported three clusters (i.e., cluster 1: “active, binge drinkers with healthy dietary intake”, cluster 2: “non-active moderate-smokers and non-drinkers with poor dietary intake” and cluster 3: “moderately active, non-smoking and non-drinkers with moderately healthy dietary intake”) using Latent Class Analysis (LCA) [39]. Finally, Tomczyk et al. reported three clusters (i.e., “low users” characterised by students who reported low use of cigarettes, alcohol and cannabis/marijuana, “alcohol users” comprised of students who reported high use of alcohol and average scores for smoking cigarettes and “polysubstance users” characterised by students who reported high use of cigarette smoking, alcohol use and cannabis/marijuana) using Latent Transition Analysis (LTA) [40].

### 3.5. Socio-Demographic Characteristics Associated with Co-Occurrence of Multiple Health Risk Factors and/or Identified Clusters

#### 3.5.1. Gender

One study reported that females were less likely to engage in (a) hazardous drinking and tobacco smoking but more likely to engage in (b) hazardous drinking and physical inactivity; (c) tobacco smoking and physical inactivity and (d) hazardous drinking, tobacco smoking and physical inactivity [42]. All three studies that examined the clustering patterns of health risk factors did not find an association between gender and the identified clusters [35,39,40].

#### 3.5.2. Age

One study reported that vocational education students aged 17 years and older were more likely to engage in hazardous drinking and physical inactivity whereas those aged 21 years or more were more likely to smoke tobacco and be physically inactive [42]. Participants older than 18 years were more likely to engage in hazardous drinking, tobacco smoking and physical inactivity [42].

The study by Jeffries et al. reported that for every additional year in age, participants were 8% more likely to belong to the “active, binge drinkers with healthy dietary intake” cluster [39]. Tomczyk et al. did not find any association between age and the clusters reported in their study [40] while Bannink and colleagues reported no association between age and the substance use cluster [35].

#### 3.5.3. Socio-Economic Status (SES)

Only one study examined whether SES was associated with clusters and found no significant association between SES and “low users,” “alcohol users” or “polysubstance users” [40].

#### 3.5.4. Education

Haug et al. found that those with secondary school education were less likely than those with no educational qualification to engage in hazardous drinking and tobacco smoking [42]. The remaining studies did not examine whether level of education was associated with engaging in multiple health risk factors or clusters [35,36,37,38,39,40,41].

#### 3.5.5. Ethnicity

Bannink et al. reported that participants of non-Dutch ethnicity were less likely to belong to the “substance use” cluster [35]. In the study by Haug et al. participants who had both parents born outside Switzerland were less likely than those with none of their parents born outside of Switzerland to engage in (a) hazardous drinking and tobacco smoking and (b) hazardous drinking and physical inactivity but more likely to engage in tobacco smoking and physical inactivity [42].

### 3.6. Methodological Quality Assessment

Table 3 outlines the methodological quality of the eight included studies in relation to 14 items and the overall quality rating. In terms of the overall quality rating one study was rated as good [40], six studies were rated as fair [35,36,38,39,41,42] and one study was rated as poor [37]. All studies adequately reported the research question and reported their participation/consent rate to be above 50%. Six studies [36,38,39,40,41,42], adequately reported the study population using demographics, locations, and time period whereas two studies [35,37] did not clearly specify and define the study population. Seven studies reported the eligibility criteria used to recruit the sample [35,36,37,38,39,41,42]. None of the studies provided a justification for the sample size. Five studies [35,37,39,40,42] did not report if outcome assessors were blinded and the longitudinal study did not report the follow-up rate [40]. Four studies did not provide information to indicate whether potential confounding factors were measured and adjusted for [36,37,38,41]. We could not determine if the outcome measures used were validated for six studies [35,36,38,40,41,42]. None of the studies measured the exposure variables more than once. Seven studies did not assess the exposure variables before the outcome variables were measured and did not allow a sufficient timeframe to see any effect [35,36,37,38,39,41,42].

## 4. Discussion

This is the first systematic review to examine the co-occurrence of multiple health risk factors, clustering of health risk factors and socio-demographic characteristics associated with co-occurrence of multiple health risk factors and/or identified clusters among vocational education students. Eight studies were included in the review. Five studies [36,37,38,41,42] reported the co-occurrence of health risk factors but only one of these studies reported the socio-demographic characteristics associated with co-occurrence of health risk factors [42]. Three of the eight studies examined the clustering of health risk factors and socio-demographic characteristics associated with cluster membership [35,39,40].

Across five studies, vocational education students’ engagement in two or more health risk factors ranged from 29% [42] to 98% [36]. This evidence highlights that a substantial proportion of vocational education students engage in multiple health risk factors. However, there were no studies that included all of the eight health risk factors examined in this review. All eight studies measured alcohol consumption [35,36,37,38,39,40,41,42]; seven studies measured smoking cigarettes/tobacco [35,36,38,39,40,41,42]; three measured physical activity [36,39,42]; two measured depression and/or anxiety [35,37]; two measured obesity/overweight [36,39], and one study measured fruit and vegetable consumption [36]. This highlights that substance use factors were more frequently reported than other health risk factors among vocational education students. Future studies should therefore take a broader approach to measuring the co-occurrence of multiple health risk factors in vocational education students by including a more comprehensive list of factors related to physical and mental health.

The three studies that reported the clustering of health risk factors reported clustering of alcohol use and tobacco smoking among vocational education students [35,39,40]. This is consistent with previous studies among university students and adult populations that have reported a strong clustering pattern of tobacco smoking and alcohol use [7,29]. Other health risk factors found to cluster together were (i) physical activity, binge drinking, and healthy diet [39] and (ii) physical inactivity, smoking and poor diet [39]. This emphasises the need to take a holistic approach to behaviour change in the vocational education setting. Multiple health risk factor interventions that address health risks simultaneously or sequentially may be particularly beneficial for vocational education students. However, importantly none of the existing studies examined clustering of all eight health risk factors together in vocational education students. Future research should assess how all eight health risk factors cluster together in vocational education students in order to inform the development and delivery of effective, comprehensive preventive health interventions in the vocational education setting. Clustering between physical activity and alcohol consumption has not received much attention with regards to policy. Further research should explore this relationship.

Only four [35,39,40,42] of the eight studies examined socio-demographic characteristics associated with the co-occurrence of multiple health risk factors or clustering of health risk factors. Gender and age were examined as characteristics that may be associated with co-occurrence of health risk factors or clusters in all four studies [35,39,40,42]. In relation to gender the findings were mixed with only one of the four studies reporting an association between gender and co-occurrence of health risk factors [42]. Specifically, females were found to be (i) less likely to engage in hazardous drinking and tobacco smoking but more likely to engage in (ii) hazardous drinking and physical inactivity, (iii) tobacco smoking and physical inactivity and (iv) hazardous drinking, tobacco smoking and physical inactivity. In terms of age, two of the four studies reported a significant association, specifically Haug and colleagues reported that participants aged 17 years and above were more likely to engage in hazardous drinking and physical inactivity, participants aged 21 years and above were more likely to report they smoked tobacco and physical inactivity and those aged 19 years and older were more likely to engage in hazardous drinking, tobacco smoking and physical inactivity [42]. In the study by Jeffries et al. for every additional increase in age, participants were more likely to belong to the ‘active, binge drinkers and healthy dietary intake’ cluster [39]. The findings by Haug et al. [38] are consistent with previous studies [43,44,45] that reported females were more likely to belong to clusters characterised by physical inactivity, hazardous drinking, and tobacco smoking among young adults in universities. Further research is needed among vocational education students to strengthen the evidence-base about potential associations between gender, age, SES, education and ethnicity and the co-occurrence of health risk factors or clustering of multiple health risk factors.

Given that vocational education settings have a large number of students, they are an ideal setting for targeting multiple health risk factors [17]. Vocational education settings also have facilities such as gyms which could be helpful for modifying health risk factors [46]. Furthermore, vocational education students are often adolescents or young adults [17] which provides opportunity to modify health risk factors earlier in life in an effort to produce positive health outcomes in the short- and long-term.

### Limitations

This systematic review has a number of limitations. Firstly, all studies defined health risk factors differently and measured these health factors using different measures. This contributed to the heterogeneity in this review. Secondly, all three studies that reported clustering of health risk factors used different statistical techniques to identify clusters. The different statistical techniques used to identify clusters makes comparison between studies challenging. Thirdly, some studies we excluded measured more than one health risk factor but failed to analyse and report the co-occurrence or clustering of multiple health risk factors. This illustrates that health risk factors in vocational education students are often considered in isolation rather than a holistic approach adopted of analysing multiple health risk factors collectively. Finally, the methodological quality assessment was based on information available in the published article and it is possible that inadequate reporting influenced the ratings.

## 5. Conclusions

This systematic review identified limited high-quality evidence about the co-occurrence of multiple health risk factors, clustering of health risk factors and associated socio-demographic characteristics in vocational education students. Given that no studies examined all eight health risk factors associated with NCDs, further research is needed to conduct a more comprehensive assessment of how these multiple health risk factors co-occur or cluster in vocational education students. This could inform the design of multiple health risk interventions that holistically target vocational education students’ health risk behaviours and mental health concurrently instead of targeting them in isolation.

## Figures and Tables

**Table 1:** Search strategy.

#	Searches
1	smoke/or smoking cessation/or smoking/or cigarette smoking/or smok*.mp. or tobacco/
2	smoking.mp. or smoking/
3	1 or 2
4	nutrition/or nutrition.mp.
5	unhealthy diet/or diet.mp. or healthy diet/or diet/
6	fruit*.mp. or fruit/
7	vegetable/or vegetable*.mp.
8	4 or 5 or 6 or 7
9	Alcohol*.mp. or alcohol intoxication/or alcohol/ or alcohol consumption/
10	binge drinking.mp. or drinking behavior/ or binge drinking/
11	9 or 10
12	exercise/or physical activity/or physical activ*.mp.
13	physical inactivity/or physical inactiv*.mp.
14	sedentary lifestyle/or sedentary*.mp.
15	12 or 13 or 14
16	obesity/or obesity.mp.
17	body weight.mp. or body weight/
18	weight loss.mp. or body weight loss/
19	weight control.mp. or body weight control/
20	overweight.mp.
21	body mass index.mp. or body mass/
22	16 or 17 or 18 or 19 or 20 or 21
23	multiple health risk behavio?r*.mp.
24	multiple behavio?r*.mp.
25	multiple risk behavio?r*.mp.
26	multiple lifestyle behavio?r*.mp.
27	multiple health behavio?r*.mp.
28	23 or 24 or 25 or 26 or 27
29	mental health.mp. or mental health/
30	psychological distress.mp.
31	depression/or depression.mp.
32	anxiety.mp. or anxiety/
33	29 or 30 or 31 or 32
34	vocational education.mp. or vocational education/
35	technical education.mp.
36	polytechnic*.mp.
37	apprentice.mp.
38	Technical Schools.mp.
39	community colleges.mp.
40	further education.mp.
41	vocational education and training.mp.
42	Technical and Vocational Education Training.mp.
43	vocational training.mp.
44	TAFE.mp.
45	34 or 35 or 36 or 37 or 38 or 39 or 40 or 41 or 42 or 43 or 44
46	3 and (8 or 11 or 15 or 22 or 33)
47	8 and (3 or 11 or 15 or 22 or 33)
48	11 and (3 or 8 or 15 or 22 or 33)
49	15 and (3 or 8 or 11 or 22 or 33)
50	22 and (3 or 8 or 11 or 15 or 33)
51	33 and (3 or 8 or 11 or 15 or 22)
52	46 or 47 or 48 or 49 or 50 or 51
53	28 or 52
54	53 and 45

* Different forms of words or plurals.

**Table 2:** Characteristics of studies examining the co-occurrence and clustering of health risk factors.

Author, Country, Year of Data Collection	(i) Study Design (ii) Setting	Sample Characteristics	(i) Recruitment Method (ii) Eligibility Criteria (iii) Response Rate	Measures Used	Co-Occurrence of Health Risk Factors	Description of Clusters Identified	Characteristics Associated with (i) Co-Occurrence of Health Risk Factors (ii) Clusters
Bannink et al. 2015 [35], Netherlands, 2012	(i) Cross sectional (ii) 44 first-year classes from the two lowest levels of vocational education setting	N = 584 students Mean age = 18.3 ± 2.59 years 38.9% boys 27.9% Dutch 10.6% had a child	(i) Study information received by all students and parents a few weeks prior to study commencement (ii) Two lowest levels of vocational education (the easiest levels) (iii) 70.4%	Cigarette smoking: Frequency of smoking at the time of assessment (no smoking to every day) Alcohol use: number of alcoholic drinks consumed on a single occasion in the past 4 days Depression: Frequency of depressive symptoms using the Centre for Epidemiologic Studies Depression Scale (CES-D) * Frequency of cannabis use in the past 4 weeks also measured which is included in the co-occurrence of health risk factors results	18.1% used two substances 11.0% used three substances.	2 clusters: 1. “substance use” cluster: Binge drinking, cannabis use and cigarette smoking 2. “problem behaviour” cluster: delinquency, truancy and incurring debts	(i) Not reported (ii) Ethnicity and Depressive symptoms associated with “substance use” cluster
Bonevski et al. 2013 [36], Australia, 2010	(i) Cross sectional (ii) One Technical and Further Education (TAFE) campus	N = 224 Age = 16 years and above 51.3% females 67.1% Australian-born, 80% spoke English at home, 55.6% lived with parent/guardian, 46.3% earned < $300 as personal weekly income, 59% attended TAFE full time	(i) Identified key contact distributed information letters to consenting teachers to give to eligible students 1 week before survey administration. (ii) Classes with high number of English-speaking students, on-campus classes and a high proportion of younger students (16–24 years). (iii) 97%	Smoking: Current smoking status Fruit and vegetable consumption: Number of daily fruit and vegetable serves Alcohol use: Number of standard drinks consumed on one occasion. Physical activity: Total times spent engaging in moderate and vigorous physical activity Body Mass index: Calculated based on self-reported height and weight * Sun tanning behaviour also measured which is included in the co-occurrence of health risk factors results	98% reported two or more health risk behaviours.	Not reported	Not reported
Cadigan and Lee, 2019 [37], USA, not stated	(i) Cross sectional (ii) Three public community colleges	N = 142 Mean age = 22.75 ± 3.34 years 69.7% female 58.9% White 12.8% Asian 5.7% Black 14.9% multiracial 2.8% American Indian/Alaskan Native, 2.8% Native Hawaiian/Pacific Islander, 2.1% other ethnicity	(i) Displayed posters and distributed handouts at campuses, ads in college newspapers and emails. (ii) 18- to 29-year-olds, enrolled full or part time at community colleges, engaged in heavy drinking (i.e., 4+/5+ drinks for women and men respectively or exceeding weekly National Institution on Alcohol Abuse and Alcoholism drinking recommendations of 8+/15+ for women and men respectively) and owned/used a cell phone (iii) 90%	Alcohol use: Alcohol Use Disorders Identification Test (AUDIT) to measure the quantity and frequency of alcohol use and consequences of alcohol use. Depression: Depressive symptoms assessed using the Patient Health Questionnaire (PHQ-8) Anxiety: Anxiety symptoms assessed using the Generalized Anxiety Disorder-7 (GAD-7)	21.8% depression and anxiety 32% heavy drinking and depression 25% heavy drinking and anxiety	Not reported	Not reported
Chyderiotis et al. 2020 [38], France, 2008 and 2017	(i) Cross- sectional (ii) Vocational students and apprentices	2008 n = 4564 apprentices Mean age = 17.4 years 2017 sample A n = 877 apprentices Mean age = 17.4 years 73.7% boys 54.9% living with parents 53.1% grade repetition 2017 Sample B N = 949 Mean age = 17.4 years 73.8% boys 54.5% living with parents 55.8% grade repetition	(i) All adolescents who attended the National Defence and Citizenship Day (JDC) were invited to participate. (ii) Apprentices aged 17 in 2008 and 2017 (iii) >90% in 2008 and 2017	Cigarette smoking: Smoking during the last 30 days. Alcohol use: Alcohol use in the past month	Sample A 2008 54.6% daily smoking and alcohol use in the past month 2017 sample A 51.1% daily smoking and alcohol use in the past month Sample B 2017 52.1% daily smoking and alcohol use in the past month	Not reported	Not reported
Haug et al. 2013 [42], Switzerland, 2011-2012	(i) Cross-sectional (ii) 24 post-secondary vocational education campuses	(i) N = 2647 Median age = 18 years 50.9% females 78.8% had secondary school education 53.8% no immigrant background	(i) All students in participating classes invited by externally trained staff to participate in the survey during a health education lesson. (ii) Not stated (iii) 99.5%	Tobacco smoking: daily /occasional smokers Alcohol use: quantity consumed, frequency of consumption and binge drinking in the previous year Physical activity: hours spent engaging in moderate to vigorous physical activity in a week	34% had two health risk factors 24.5% hazardous drinking and tobacco smoking 6.4% hazardous drinking and physical inactivity 3.1% tobacco smoking and physical inactivity 9.6% had all three health risk factors	Not reported	(i) Gender (ii) Age (iii) Education (iv) Ethnicity
Jeffries et al. 2018 [39], USA, 2011 and 2012	(i) Cross sectional (ii) Three 2-year community colleges	N = 441 Mean age 22.7 ± 5 years 67.6% female 72.6% white Average BMI = 25.4 ± 3.8 54.4% living with parent	(i) Not stated (ii) Enrolled in the Choosing Healthy Options in College Environments and Settings (CHOICES) study and completed baseline assessments. (iii) 99.5%	Tobacco smoking: daily/occasional smokers Alcohol use: Binge drinking within the past 30 days Physical activity: Total minutes spent engaging in moderate-intensity aerobic activity in a week Body mass index: Height (metres) and weight (kg) measured by trained staff using height boards and scales.	Not reported	**3 classes** Class 1 (13.1%): active, binge-drinkers with a healthy dietary intake Class 2 (38.2%): non-active, moderate-smokers and non-drinkers with poor dietary intake Class 3 (48.7%): moderately active, non-smoking and non-drinkers with moderately healthy dietary intake	(i) Body mass index (BMI) associated with class 2 membership Age associated with class 1 membership
Tomcyzk et al. 2016 [40] Germany Year of data collection not reported	(i) Longitudinal (ii) 49 vocational schools in seven German states	N = 5688 54% males Mean Age = 19.39 ± 3.92 years	(i) Not stated (ii) Not stated (iii) 79%	Substance use: frequency of use of alcohol and cigarettes * Other substances measured included: Cannabis/marijuana	Not reported	**3 classes** Class 1 (43%) “low users” had low scores on all substance use Class 2 (50%) “alcohol users” had students with high scores for alcohol use and average scores on cigarette use Class 3 (7%) “polysubstance use” had students with high scores on all substance use	Job stress associated with class 3.
Underwood et al. 2007 [41], United Kingdom, 2000 and 2005	(i) Cross-sectional (ii) 77 Vocational dental practitioners (VDP)	2000 N = 534 2005 N = 502 No sample characteristics were reported.	(i) Questionnaire packs were sent to all UK VDP advisors, for distribution to their VDP groups at their next study day. (ii) Not reported (iii) 75% in 2000 and 66% in 2005	Frequency and amount of tobacco smoking and alcohol use	55% smoked tobacco and drank alcohol	Not reported	Not reported

* = Other risk behaviours measured by the included studies but were not the focus of this review.

**Table 3:** Methodological quality assessment of eligible studies.

	Q1 Research Question	Q2 Study Population	Q3 Participation/Response Rate	Q4 Groups Recruited From the Same Population and Uniform Eligibility Criteria	Q5 Sample Size Justification	Q6 Exposure Assessed Prior to Outcome Measurement	Q7 Sufficient Timeframe to See an Effect	Q8 Different Levels of the Exposure of Interest	Q9 Exposure Measures and Assessment	Q10 Repeated Exposure Assessment	Q11 Outcome Measures	Q12 Blinding of Outcome Assessors	Q13 Follow-Up Rate	Q14 Measurement and Adjustment of Potential Confounders	Quality Rating
Bannink et al. [35]	Yes	No	Yes	Yes	No	No	No	Yes	Yes	No	CD	NR	NA	Yes	Fair
Bonevski et al. [36]	Yes	Yes	Yes	Yes	No	No	No	Yes	CD	No	CD	NA	NA	No	Fair
Cadigan et al. [37]	Yes	No	Yes	Yes	No	No	No	NA	NA	No	Yes	NR	NA	No	Poor
Chyderiotis et al. [38]	Yes	Yes	Yes	Yes	No	No	No	Yes	CD	No	CD	NA	NA	No	Fair
Haug et al. [42]	Yes	Yes	Yes	Yes	No	No	No	Yes	CD	No	CD	NR	NA	Yes	Fair
Jeffries et al. [39]	Yes	Yes	Yes	Yes	No	No	No	Yes	CD	No	Yes	NR	NA	Yes	Fair
Tomcyzk et al. [40]	Yes	Yes	Yes	No	No	Yes	Yes	Yes	Yes	No	CD	NR	No	Yes	Good
Underwoood et al. [41]	Yes	Yes	Yes	Yes	No	No	No	NA	NA	No	CD	NA	NA	No	Fair

NA = Not applicable; CD = cannot determine; NR = Not reported.

## Data Availability

No new data were created or analysed in this study. Data sharing is not applicable to this article.
